# Structural and Functional Significance of the Endoplasmic Reticulum Unfolded Protein Response Transducers and Chaperones at the Mitochondria–ER Contacts: A Cancer Perspective

**DOI:** 10.3389/fcell.2021.641194

**Published:** 2021-03-26

**Authors:** Giuseppina Amodio, Valentina Pagliara, Ornella Moltedo, Paolo Remondelli

**Affiliations:** ^1^Department of Medicine, Surgery and Dentistry “Scuola Medica Salernitana,” University of Salerno, Baronissi, Italy; ^2^Department of Pharmacy, University of Salerno, Fisciano, Italy

**Keywords:** unfolded protein response, mitochondria–ER contacts, endoplasmic reticulum, molecular chaperones, cancer

## Abstract

In the last decades, the endoplasmic reticulum (ER) has emerged as a key coordinator of cellular homeostasis, thanks to its physical interconnection to almost all intracellular organelles. In particular, an intense and mutual crosstalk between the ER and mitochondria occurs at the mitochondria–ER contacts (MERCs). MERCs ensure a fine-tuned regulation of fundamental cellular processes, involving cell fate decision, mitochondria dynamics, metabolism, and proteostasis, which plays a pivotal role in the tumorigenesis and therapeutic response of cancer cells. Intriguingly, recent studies have shown that different components of the unfolded protein response (UPR) machinery, including PERK, IRE1α, and ER chaperones, localize at MERCs. These proteins appear to exhibit multifaceted roles that expand beyond protein folding and UPR transduction and are often related to the control of calcium fluxes to the mitochondria, thus acquiring relevance to cell survival and death. In this review, we highlight the novel functions played by PERK, IRE1α, and ER chaperones at MERCs focusing on their impact on tumor development.

## Introduction

The endoplasmic reticulum (ER) presides over the biogenesis and maintenance of almost all the cell compartment and participates in the regulation of their functions throughout exchanges of structural molecules and signaling factors. It is well known that the ER is the place where lipid and steroids are synthesized and, then, delivered to other endo-membranes throughout vesicular carriers or non-vesicular mechanisms that, if out of control, result in several pathologies including cancer (Peretti et al., 2019). It is universally recognized that the largest part of the intracellular calcium is stored within the ER and that different ER integral membrane proteins control Ca^2+^ homeostasis by modulating either the ion uptake or its delivery to the neighboring compartments (Putney, 2005; Clapham, 2007). Remarkably, calcium is engaged in the managing of several pathophysiological processes having an important impact in human malignancies development (Limia et al., 2019; Reich et al., 2020).

On the other hand, a large portion of the ER is associated to protein synthesis and translocation. The ER lumen is equipped with a molecular machinery that carries out a rigorous chaperone-mediated quality control (QC) of protein folding to ensure that proteins are delivered in a functional state to secretory compartments. QC is under the regulation of the unfolded protein response (UPR): a battery of signaling pathways that, when excessive protein unfolding occurs is able to choose between cell death and survival, this choice affecting either normal or cancer cells. This is why the participation of the UPR in tumorigenesis has long been studied. Moreover, the UPR has always been considered a strategic target for potential cancer therapy for its consequence on neoplastic cell proliferation and survival (Wang and Kaufman, 2014). Between the mitochondria and the ER, signals and molecules are rapidly exchanged throughout the molecular structures known as mitochondria–ER contacts (MERCs), also called mitochondria-associated membranes (MAMs) (Vance, 2014). A growing evidence shows that both UPR transducers and chaperones are structural components involved in various ways in the MERCs functions (Ilacqua et al., 2017). In this review, we aim to outline the role of UPR actors in the MERCs functions and to make a point on the consequence of their presence in MERCs in the fate of cancer cells.

## The UPR Signaling Pathway

The control of lipid and protein flux toward the secretory pathway compartments is an essential function of the ER, which is the site of entrance, folding, and departure of luminal and integral membrane proteins destined to the ER itself, Golgi apparatus, endo-lysosomal compartment, the plasma membrane (PM), or the extracellular environment (Walter and Schultz, 1981; Jan et al., 2014; Fasano et al., 2018).

Quantitatively, ER exit relies on an autoregulatory system consisting of a set of signals that, in case of overload of newly synthesized cargo proteins, modulate protein export by regulating their packaging at the ER exit sites (ERESs) into COPII anterograde vesicles and by attenuating protein synthesis to prevent traffic congestion of cargo proteins (Subramanian et al., 2019).

**FIGURE 1:**
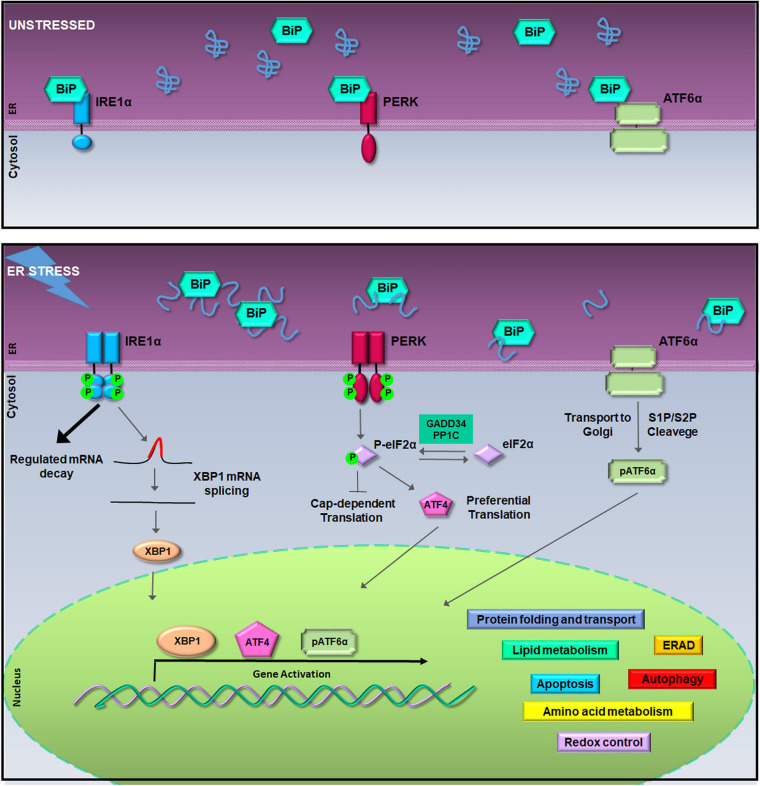
The unfolded protein response (UPR) signaling pathway. In unstressed conditions (upper box), the activation of inositol-requiring enzyme 1a (IRE1α), pancreatic ER kinase (PERK), and activating transcription factor 6a (ATF6α) is inhibited by the binding of Bip/GRP78. During the endoplasmic reticulum (ER) stress, binding of glucose regulated protein 78-kDa/binding immunoglobulin protein (GRP78/BiP) to misfolded proteins allows the activation of IRE1α, PERK, and ATF6α (lower box). Activated IRE1α cleaves 26-nucleotides from the X-box binding protein 1 (XBP1) mRNA allowing the translation of XBP1; hyper-oligomerized IRE1α executes regulated IRE1a-dependent decay (RIDD) activity on selected cytosolic mRNAs (Cox and Walter, 1996; Hollien et al., 2009). Activated PERK phosphorylates the eukaryotic initiation factor 2a (eIF2α) leading to attenuation of protein synthesis and to the preferential translation of activating transcription factor 4 (ATF4) mRNA (Harding et al., 2000). ATF6α activation is achieved in the Golgi complex where it undergoes to intramembrane proteolysis-specific cleavage by site-1 protease (S1P) and S2P to produce a transcriptionally active fragment (pATF6α). XBP1, ATF4, and pATF6α are responsible for the execution of the UPR transcriptional program in the nucleus (Walter and Ron, 2011).

Qualitatively, within the ER, a sophisticated machinery of enzymes and chaperones has evolved to retain unfunctional proteins within the organelle or to direct them to ubiquitin/proteasome degradation (Hartl and Hayer-Hartl, 2009; Ruggiano et al., 2014). QC of protein folding is controlled by the UPR pathways made of a battery of ER transmembrane transducers consisting of the pancreatic ER kinase (PERK), the inositol-requiring enzyme 1 (IRE1), and the activating transcription factor 6 (ATF6) (Schroder and Kaufman, 2005). UPR activation occurs because, normally, each transducer is kept inactive by the chaperone glucose-regulated protein 78-kDa/binding immunoglobulin protein (GRP78/BiP). Instead, when unfolded proteins increase within the ER lumen, GRP78/BiP releases IRE1, PERK, and ATF6 synchronizing their activity (Figure 1). The UPR orchestrates a vigorous expression of ER enzymes and chaperones such as calreticulin (CLR), calnexin (CLNX), GRP78/BiP, and the ER Protein 57-kDa (ERp57) with the purpose to potentiate QC machinery (Sitia and Braakman, 2003; Amodio et al., 2009). Moreover, under ER stress, the UPR activates genes encoding vesicular trafficking factors and limitates the ER exit of secretory proteins (Renna et al., 2006; Amodio et al., 2009, 2011). Furthermore, the UPR boosts up the clearance of uncorrectly folded proteins, which would otherwise compromise cell functions, by fine tuning either the ER-associated protein degradation (ERAD) (Meusser et al., 2005) or autophagy (Simmen and Herrera-Cruz, 2018).

At the same time, to limit the amount of unfolded proteins entering the ER, PERK phosphorylates the eukaryotic initiation factor 2α (eIF2α) producing p-eIF2α that acts as a suppressor of protein translation initiation (Harding, 1999; Zhao and Ackerman, 2006). This event reduces protein translocation into the ER initiating the ER folding machinery. In fact, besides the reduction of protein load, p-eIF2α allows the selective translation of a subset of mRNAs (Ma and Hendershot, 2003). Among them, activating transcription factor 4 (ATF4) is preferentially translated to regulate an adaptive response that activates transcription of ER chaperones and ER stress response genes, such as GRP78/BiP, and of genes involved in the amino acid metabolism, mitochondrial function (Ameri and Harris, 2008), autophagy (Rouschop et al., 2010), and in the oxidative stress response (Harding et al., 2000; Blais et al., 2006; Walter and Ron, 2011) (Figure 1). In particular, the ATF4 antioxidant program culminates with the PERK-dependent activation of Nrf2, a transcription factor whose target genes are involved in the antioxidant defense (Cullinan and Diehl, 2006). However, aside from PERK, eIF2α phosphorylation is carried out by other kinases such as the protein kinase R (PKR), general control non-derepressible 2 (GCN2), and heme-regulated inhibitor (HRI), which work as transducers of the stress signaling known as the integrated stress response (ISR) (Pakos-Zebrucka et al., 2016).

On the other hand, when ER stress is persistent, the UPR survival project turns into the cell death program (Zhang and Kaufman, 2006; Mori, 2009; Diehl et al., 2011; Walter and Ron, 2011) switching the PERK-eIF2α-ATF4 signaling to promote the activation of the transcription factor C/EBP homologous protein (CHOP) (Urra et al., 2013). Under these circumstances, both CHOP and ATF4 cooperate to induce amino acid biosynthetic pathways that revert translational repression, promote oxidative stress, and the expression of pro-apoptotic factors thereby leading to cell death (Marciniak et al., 2004; Han et al., 2013; Urra et al., 2013). Instead, the PERK–CHOP/growth arrest- and DNA damage-inducible gene 153 (GADD153) route is crucial for the apoptosis initiation (Zinszner et al., 1998; Rutkowski et al., 2006). This happens by activating proapoptotic factors that drive apoptosis, such as the death receptor 5 (DR5), the Tribbles ortholog in humans (Trb3), Bcl-2-interacting mediator of cell death (BIM), and P53-upregulated modulator of apoptosis (PUMA) (Yamaguchi and Wang, 2004; Ohoka et al., 2005; Puthalakath et al., 2007; Cazanave et al., 2010). Notably, CHOP also induces the regulatory subunit of GADD34, which dephosphorylates eIF2α and initiates a negative feedback that restores mRNA translation and protein synthesis. Moreover, CHOP and GADD34 knockout preserves tissues from ER stress-mediated damage, intensifies protein misfolding, and leads to apoptosis (Marciniak et al., 2004).

A similar shift from pro-survival to pro-apoptotic activity happens to IRE1α transducer, which owns both kinase and RNAse activity (Wang et al., 1998). During ER stress, freed from GRP78/BiP, IRE1α undergoes sequential autophosphorylation, conformational change, and higher order oligomerization that activates its RNase domain (Korennykh et al., 2009; Gardner and Walter, 2011). The main target of IRE1α RNase activity is the X-box-binding protein 1 (XBP1) mRNA, whose splicing allows translation of the XBP1 transcription factor that, once in the nucleus, upregulates genes implicated in the QC and cell survival, including ER chaperones and ERAD factors (Cox and Walter, 1996). However, during persistent ER stress, IRE1α hyper-oligomerization enhances RNase activity of specific mRNAs encoding ER resident proteins, an event known as regulated IRE1α-dependent decay (RIDD) (Hollien et al., 2009; Ghosh et al., 2014) (Figure 1). Therefore, RIDD activity first promotes cell survival by limiting the number of proteins entering the ER, but then, during irresolvable ER stress, it favors apoptosis through the degradation of anti-apoptotic microRNAs, for instance, those that control TXNIP and caspase-2 expression (Lerner et al., 2012; Upton et al., 2012). Thus, RIDD activity along with the TRAF2–ASK1–JNK pathway activation, also due to enhanced kinase activity of hyper-oligomerized IRE1α, switches the IRE1α signaling from survival to apoptosis during unresolved ER stress (Urano et al., 2000; Nishitoh et al., 2002).

It is universally acknowledged that the UPR plays critical roles in tumor progression, survival, and metastasis (Romero-Ramirez et al., 2004; Lee, 2007; Moenner et al., 2007). Moreover, in cancer cells, the UPR is often activated, and ER chaperones are overexpressed in response to various environmental conditions including hypoxia, oxidative stress, and nutrient starvation (Blais et al., 2006; Luo and Lee, 2013). On these grounds, and based on growing evidences showing that UPR transducers and their related chaperones are involved in various ways in the MERCs structure, we will discuss the functional significance of their residence at MERCs and their role in the pathogenesis of cancer.

## The Membrane Contact Sites

The ER shows a complex membrane architecture consisting of plane sheets in continuity with the nuclear envelope (NE) and peripheral membranes made of tubules that owe their shape to endogenous, or also exogenous, proteins that form and stabilize tubules at the ER sheet boundaries (Shibata et al., 2010; Friedman et al., 2011; Voeltz and Barr, 2013; Westrate et al., 2015; Grimaldi et al., 2018). While ER sheets are involved in protein synthesis, the ER tubular network is free of ribosomes and extremely dynamic, as it rapidly expands and eventually re-modulates by repetitive fission and fusion events (Lee and Chen, 1993). Tubules may efficiently move since they are interconnected with microtubules throughout motor proteins (namely, kinesins and dyneins) and, thereof, they can reach almost all the cell components thus facilitating the development of several connections known as membrane contact sites (MCS) (Phillips and Voeltz, 2016). MERCs were the first inter-organelle connections discovered (Bernhard and Rouiller, 1956; Vance and Shiao, 1996) followed by ER–PM (Stefan et al., 2013), ER–Golgi (Hanada et al., 2003; Peretti et al., 2008), ER–peroxisomes (Costello et al., 2017), and ER–lipid droplets (LDs) (Walther et al., 2017) disclosing that organelles rely on MCSs for many interdependent functions. MCSs are distributed along ER membrane tubules and are overall stable, since they are preserved during trafficking and membrane fusion and fission (Wu et al., 2018).

Findings on MCSs overturned the view of the cell seen like a complex of isolated compartments and acknowledged the ER, the role of intermembrane network that homeostatically controls exchange of several structural and bioactive molecules (Jan et al., 2014; Phillips and Voeltz, 2016). Despite this, so far, little is still known about the role of MCSs in pathogenic processes including cancer.

Mitochondria–ER contacts are spaced apart from 10 to 80 nm (Krols et al., 2016; Sezgin et al., 2017; Chakkarapani et al., 2018; Moltedo et al., 2019) suggesting that the two organelles are kept together by tethering factors, holding the two opposite membranes in close proximity. Among them is Mitofusin 2 (Mfn2), a large GTPase usually involved in outer mitochondrial membrane (OMM) fusion events. Mnf2 interacts in trans with Mfn1 or Mfn2 forming hetero- or homo-dimers that hook mitochondria to the ER. Another tethering complex is formed by the vesicle-associated protein B (VAPB), an ER membrane protein that interacts with the OMM protein tyrosine phosphatase-interacting protein-51 (PTPIP51). In addition, at MERCs, the ER calcium-release channel inositol 1,4,5-trisphosphate receptor (IP3R) interacts with the voltage-dependent anion channel (VDAC) located at the OMM, through the molecular chaperone glucose-regulated protein 75-kDa (GRP75), and such a complex regulates Ca^2+^ flux from the ER to the mitochondria (Moltedo et al., 2019).

In this context, MERCs provide fundamental platforms for several cellular functions, such as phospholipid synthesis and exchange, Ca^2+^ flows, mitochondrial fission, and apoptosis. The homeostatic control of Ca^2+^ occurring at these sites has constantly attracted particular interest, and MERCs are growingly emerging as the residence of the regulation of cancer cell onset, growth, progression, and metabolism (Herrera-Cruz and Simmen, 2017). Indeed, altered Ca^2+^ signaling at the MERCs is considered a characteristic of cancer cells because it can shift cell metabolism to glycolysis and increases cancer cell resistance to cell death (Herrera-Cruz and Simmen, 2017). Cancer cells fundamentally require basal mitochondrial Ca^2+^ uptake for survival, thus lowering ER-to-mitochondria Ca^2+^ transfer causing cancer cell death (Cardenas et al., 2016). In contrast, mitochondrial Ca^2+^ overload can lead to the opening of mitochondrial permeability transition pore (PTP). As a consequence, PTP induction leads to the increase of IMM permeability and the loss of mitochondrial membrane potential, which, in turn, induces mitochondrial swelling, OMM break, cytochrome c release, and cell death (Rizzuto et al., 2012). For this reason, Ca^2+^ accumulation in the mitochondrial matrix has important implications also in other processes, including autophagy, metabolism, and apoptosis (Morciano et al., 2018). In particular, apoptosis is closely connected to calcium status of mitochondria (Csordas et al., 2006) and tumorigenesis. In addition, it has been noted that conditions that prevent massive Ca^2+^ transfer from the ER to the mitochondria through the regulation of Ca^2+^ channel activity, protect from cell death normal cells, but can lead to neoplastic transformation (Rizzuto et al., 2012). It is also worth noting that numerous regulators of Ca^2+^ flux acting as tumor suppressors and oncogenes have been recently identified as MERC proteins (Marchi and Pinton, 2014; Bittremieux et al., 2016).

Mitochondrial Ca^2+^ flux is also regulated by the mitochondrial calcium uniporter (MCU) complex (Raffaello et al., 2012; Marchi and Pinton, 2014). This channel is located at the inner mitochondrial membrane (IMM) and is composed of uniporter pore-forming subunits MCU, MCUb, and the essential MCU regulator (EMRE). Two regulatory proteins (MICU1 and MICU2) act as a gatekeeper. Normally, MICU2 keeps the channel closed, but when the Ca^2+^ level is higher, Ca^2+^ MICU2 undergoes to a conformational change and dissociates from the complex. MICU2 release produces the loss of the gatekeeping function and formation of the MICU1–MICU1 homodimer that improves mitochondrial calcium uptake (Patron et al., 2014). Interestingly, dysfunction in one or more MCU complex subunits has been associated to different cancers (Marchi et al., 2019).

## Pancreatic ER Kinase at Mitochondria–ER Contacts: Structural Role and Modulation of Cancer Progression

**FIGURE 2:**
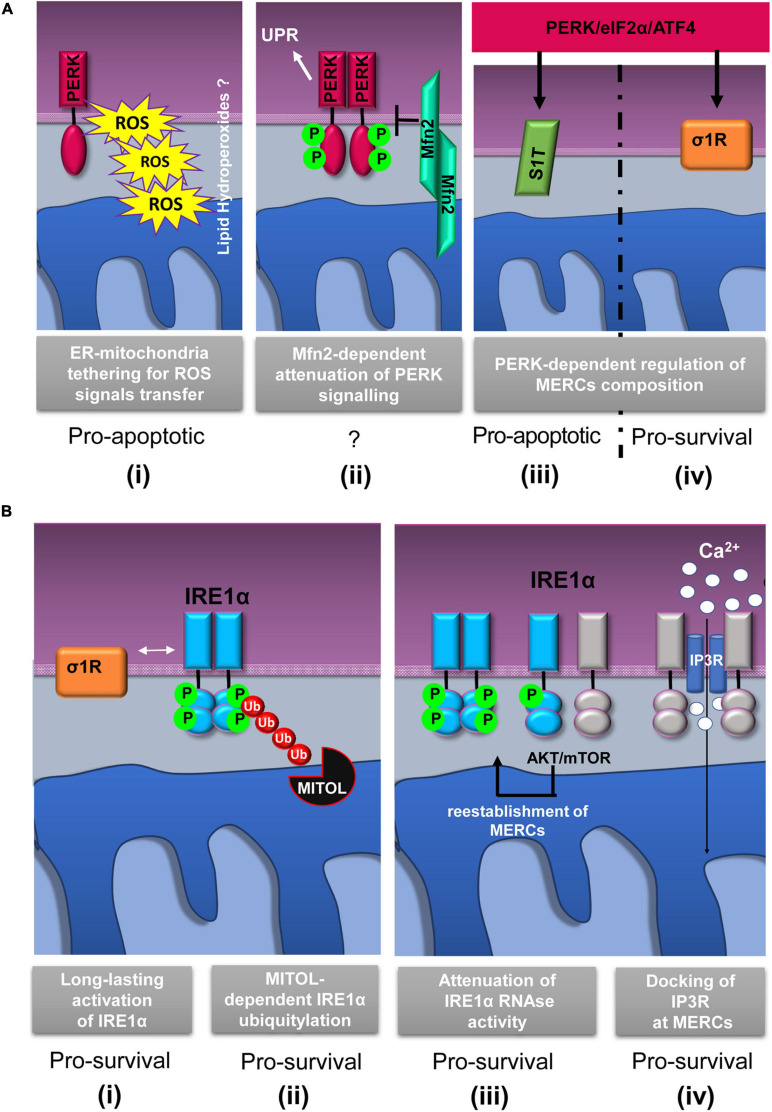
Schematic representation of relevant events involving IRE1α and PERK proteins at mitochondria–ER contacts (MERCs). **(A)** PERK-involving events at MERCs: **(i)** PERK tethers the ER to mitochondria promoting the rapid transfer of reactive oxygen species (ROS) signals, likely under the form of lipid hydroperoxides (Verfaillie et al., 2012); **(ii)** Mitofusin 2 (Mfn2) lies upstream of PERK and under basal conditions maintains PERK inactive (Munoz et al., 2013); the PERK/eIF2α/ATF4 signaling pathway upregulates the expression of SERCA1 truncated proteins (S1T) **(iii)** and sigma-1 receptor (σ1R) **(iv)** at MERCs (Chami et al., 2008; Mitsuda et al., 2011). **(B)** Events involving IRE1α at MERCs: **(i)** σ1R-dependent stabilization of IRE1α oligomerization at MERCs (Mori et al., 2013); **(ii)** mitochondrial ubiquitin ligase (MITOL)-dependent ubiquitylation of IRE1α at MERCs prevents ER stress induced apoptosis (Takeda et al., 2019); **(iii)** the AKT-mammalian target of rapamycin (mTOR) signaling attenuates IRE1 RNase activity by promoting the re-establishment of ER-mitochondria contacts (Sanchez-Alvarez et al., 2017); **(iv)** IRE1α scaffolds IP_3_R at MERCs to sustain calcium transfer and mitochondrial bioenergetics (Carreras-Sureda et al., 2019). The *putative* pro-survival or pro-apoptotic outputs of the depicted events are reported.

Since its discovery, PERK localization at MERCs has suggested a non-canonical mechanism through which this protein could communicate with mitochondria to drive cell death. In an exemplary work, it has been shown that PERK localizes at MERCs to establish structural and functional connection with mitochondria thus facilitating the propagation of ROS-based stress signals and apoptotic signals from the ER (Verfaillie et al., 2012) (Figure 2A,i). Instead, cells lacking PERK show less solid ER–mitochondria contacts, and as a consequence, cells are less sensitive to cell death in response to ER-derived ROS. In addition, these studies show that since a kinase mutant of PERK is still maintained at MERCs, the tethering function of MERC-localized PERK is independent of its UPR transducer activity (Verfaillie et al., 2012; Cao and Kaufman, 2014).

It is important to consider that ER stress can be communicated to the mitochondria through MERCs and in particular by the PERK-mediated branch of the UPR (Chami et al., 2008; Mitsuda et al., 2011; Munoz et al., 2013; van Vliet and Agostinis, 2016; Saito and Imaizumi, 2018) thanks to the PERK-induced overexpression of a truncated variant of the ER Ca^2+^ ATPase (SERCA1), known as S1T, which is located at MERCs (Figure 2A,iii). S1T upregulation at MERCs is concomitant to mitochondrial Ca^2+^ overload, increased number of MERCs, inhibition of mitochondrial dynamics, and, in the end, apoptosis (Chami et al., 2008).

Similarly, further research has shown that, under basal conditions, PERK signaling could be inhibited by the interaction to Mfn2 at MERCs (Munoz et al., 2013) (Figure 2A,ii). As proof of this, it has been observed that Mfn2 ablation causes a sustained activation of PERK in unstressed cells and that, unexpectedly, PERK silencing rescues ROS production, mitochondrial Ca^2+^ overload, and defective mitochondrial morphology in Mfn2-deficient cells. On these grounds, it is very likely that Mfn2 could act as an upstream regulator of the PERK signaling and that, strikingly, such a regulation occurs at MERCs suggesting that MERCs can be seen as hot spots for the stress signaling transmission and modulation.

Interestingly, more recently, an additional role of PERK in the control of mitochondrial functions has emerged that strengthens the importance of its localization at MERCs. Indeed, PERK is required to modulate mitochondrial morphology (Munoz et al., 2013). In particular, PERK promotes protective stress-induced mitochondrial hyperfusion (SIMH) in order to prevent mitochondrial fragmentation and favor mitochondrial metabolism in response to ER stress (Lebeau et al., 2018). Furthermore, in line with these evidence, it is reported that PERK is involved in the remodeling of mitochondrial inner membranes and in the ATF4-mediated upregulation of the super complex assembly factor (SCAF), a protein responsible for the anchoring of the respiratory chain complexes to the IMM with the effect of enhancing ATP production during ER stress (Balsa et al., 2019).

To sum up, these data suggest that PERK is involved in the control of mitochondrial bioenergetics and morphology occurring at MERCs. However, since these effects have been described in ER stress conditions or nutrient deprivation, additional studies need to be carried out in order to understand whether these effects are independent by the PERK role in the UPR.

As promoter of the antioxidant defense, the PERK pathway is widely involved in cancer initiation and progression. Indeed, the higher rate of metabolism required to support malignant expansion exposes cancer cells to excessive production of ROS and self-cytotoxic stimuli, which, for cancer cell survival, is the main obstacle to deal with. In this harmful microenvironment, cancer cells develop adaptive mechanisms to escape apoptosis, among them, the utilization of the PERK pro-survival signaling (Ma and Hendershot, 2004; Bi et al., 2005). In particular, PERK-driven Nrf2 activation runs regeneration of intracellular antioxidants, neutralization of ROS, defense from oxidative DNA damage, cell cycle checkpoint inhibition, and significant improvement of tumor growth (Luo and Lee, 2013). In most cases, cancer cells show a higher level of phospho-PERK and phospho-eIF2α (Avivar-Valderas et al., 2011; Cubillos-Ruiz et al., 2017; Feng et al., 2017; Li et al., 2018). For example, elevated levels of p-eIF2α are reported in bronchioloalveolar and gastrointestinal carcinoma, Hodgkin’s lymphoma, as well as in benign and malignant melanocytic and colon epithelial neoplasms (Lobo et al., 2000; Rosenwald et al., 2001, 2003, 2008). It has been remarked that the p-eIF2α level is correlated to a disease-free survival and better prognosis in triple negative breast cancer (TNBC) patients (Guo et al., 2017). Additionally, as demonstrated in K-Ras-transformed tumor cells, PERK can induce the expression of different pro-angiogenic factors in cells subjected to hypoxia. This event favors the formation of microvessels, hence, the adaptation of cancer cells to hypoxic stress (Blais et al., 2006). Accordingly, the PERK/ATF4 axis induces VEGF expression and, thereof, stimulates endothelial cells survival and angiogenesis in hostile microenvironments (Ghosh et al., 2010). Moreover, PERK activity is required for epithelial-to-mesenchymal transition (EMT) usually employed by cancer cells to migrate and metastasize (Denard et al., 2012; Feng et al., 2017). In particular, the PERK pathway axis mediates the upregulation of CREB3L1 in the mesenchymal subtype of TNBC resulting in enhanced metastasis and poor prognosis. In this regard, it is important to underline that the inhibition of CREB3L1 was proposed as a better therapeutic strategy since the pharmacological inhibition of PERK is known to cause severe side effects on pancreas metabolism (Feng et al., 2017).

On these grounds, by both enhancing angiogenesis and EMT, PERK is strictly correlated to cancer invasion and metastasis. Accordingly, PERK knockout reduces tumor growth and impairs angiogenesis and metastasis spread (Gupta et al., 2009). In addition, it has been shown that in de-differentiated malignant cells, the PERK/Nrf2 signaling is constitutively activated to protect from chemotherapy by reducing ROS levels and increasing drug efflux, thus implying PERK in the mechanisms of multidrug resistance (MDR) (Del Vecchio et al., 2014). Research also reports that resistant phenotypes are due to PERK/Nrf2-induced expression of the plasma-membrane transporter MDR-related protein 1 (MRP1), while disrupted PERK-to-Nrf2 axis reverses resistance to chemotherapy (Salaroglio et al., 2017). However, a large body of literature also correlates PERK signaling to antitumor activity since PERK can direct the UPR toward activation of death-triggering pathways. For an example, in human osteosarcoma (OS), one of the most common malignant tumors in children, decreased level of p-eIF2α is found, as in a recent work by Wang et al. showing that PERK enhances apoptotic OS cell death in response to lexibulin (Wimbauer et al., 2012; Wang et al., 2019). Moreover, in hepatocellular carcinomas (HCC), the induction of PERK/eIF2α/ATF4 signaling following pterostilbene treatment is associated with HCC cell death and reduced tumor growth (Yu et al., 2019). Accordingly, in human lung adenocarcinomas, paraquat induces PERK/eIF2α/ATF4 signaling leading to cancer cells apoptosis (Wang et al., 2018), and similarly, the use of PERK kinase inhibitors *in vivo* potentiates the chemioterapics in colon cancer cells (Shi et al., 2019; Wu et al., 2019). Thereby, PERK signaling, as also seen for the IRE1α pathway (see further), seems to have both pro-survival and antitumor effects in different carcinogenesis stages and types with paradoxical outcomes in cancer cells. One interesting theory has been recently proposed in melanoma cells to explain the conflicting role exerted by PERK in tumor initiation and progression (Pytel et al., 2016), whereby PERK is a “haplo-insufficient tumor suppressor,” and gene dose determines tumor-suppressive versus tumor-promoting properties in melanoma, where retention of one allele of PERK seems essential for tumor progression, opposed to the deletion of two alleles, which generates diametrically opposing results (Pytel et al., 2016).

Conclusively, the accumulating evidence of the potential therapeutic use of novel UPR inhibitors is currently paving the way for clinical trials (Cubillos-Ruiz et al., 2017; Hsu et al., 2019; Raymundo et al., 2020). In particular, since the inhibition of PERK sensitizes cancer stem cells to apoptosis (Fujimoto et al., 2016), an important aspect of these promising therapies could be the inhibition of cancer stem cell proliferation, which is responsible for post-therapy tumor relapse in oncological patients (Li et al., 2008).

However, the contribution of PERK to cancer development is still contradictory, and it is still unclear whether it promotes or, conversely, suppresses tumorigenesis. Currently, the involvement of MERC-localized PERK to the mechanisms of cancer initiation, progression, and metastasis is not as well documented in the literature, as is for MERCs-localized IRE1α (see below). Nevertheless, it can be assumed that the role played by PERK at MERCs in the transmission of ROS-derived signals between ER and mitochondria is more significant for cancer cells in the mechanism of adaptation to oxidative stress and resistance to apoptosis. The modulation of PERK activity by Mfn2 at MERCs, in basal conditions, should be further investigated in cancer cells to understand whether Mfn2-dependent deregulation of PERK activity occurs at MERCs during carcinogenesis. At the same time, the non-canonical role performed by PERK at MERCs in the control of mitochondria bioenergetics could be strategic for cancer cells to overcome nutrient deprivation and adapt to hypoxic conditions. Altogether, increased knowledge about the contribution of MERC-localized PERK in cancer initiation and progression could considerably impact human studies and clinical therapies not only for cancer cell elimination, but for many other disorders correlated to PERK such as diabetes, neurodegenerative, or heart diseases (Amodio et al., 2019; Poplawski et al., 2019).

## Dynamics of the IRE1α Activity at Mitochondria–ER Contacts and Deregulation in Cancer

Since the discovery of the IRE1α presence at MERCs (Mori et al., 2013), it is clear that crucial events of the IRE1α regulation occur at MERCs. Initial works demonstrated that the Sigma-1 receptor (σ1R) stabilizes IRE1α at MERCs allowing a long-lasting dimeric conformation to efficiently splice the XBP1 mRNA and promote cell survival in response to mitochondria-derived ROS (Mori et al., 2013) (Figure 2B,i). In addition, more recently, the MERCs’ contribution in promoting IRE1α- pro-survival signaling has been reinforced by the finding that, at MERCs, mitochondrial ubiquitin ligase (MITOL/MARCH5) (Figure 2B,ii) prevents ER stress-induced apoptosis through IRE1α ubiquitylation and degradation (Takeda et al., 2019). Previous work showed that restoration of ER–mitochondria contacts by the AKT–mTOR signaling attenuates IRE1 RNase activity, thereby favoring the recovery from ER stress (Sanchez-Alvarez et al., 2017) (Figure 2B,iii).

In the light of these results, it was proposed that MERC stabilization limits IRE1α RNase activity that could lead to cell damage and apoptosis. It is assumed that this event could occur by a “timing mechanism” and throughout different ways. First, MERC reinstallation could destabilize IRE1α oligomers by modifying ER membrane fluidity; second, localization of IRE1α at MERCs could change its redox state and alter its endonuclease activity, and third, MERC-localized PPM1L and PP2A phosphatases could dephosphorylate and deactivate IRE1α (Sanchez-Alvarez et al., 2017).

Taken together, these data highlight that IRE1α activity modulation during ER stress occurs at MERCs. At MERCs, another IRE1α function, independent of its role in the UPR, has emerged (Carreras-Sureda et al., 2019), and recently, it was suggested that MERC-located IRE1α could act as a scaffolding protein for the IP_3_Rs to support Ca^2+^ transfer and mitochondrial bioenergetics (Figure 2B,iv). This idea is in agreement with previous evidences showing that, in non-stressed condition, reduced IRE1α activity appears to induce mitochondrial ROS production and cell death by accelerating ER-to-cytosolic efflux of Ca^2+^ through IP_3_Rs (Son et al., 2014). However, the observations just discussed confirm that IRE1 function at MERCs could be independent of its classic activity of UPR transducer.

Given its role in the control of cell death or survival, IRE1α involvement in cancer initiation and progression is not surprising. Different cancers are associated to IRE1α deregulation including leukemia, glioblastoma, myeloma, prostate, and breast cancers (Lhomond et al., 2018; Logue et al., 2018; Zhao et al., 2018; Sheng et al., 2019). Altered IRE1α expression is reported in different cancer types including bladder urothelial carcinoma, glioblastoma, cervical squamous cell carcinoma, renal clear cell carcinoma, and mesothelioma (Chandrashekar et al., 2017; Tang et al., 2017). In many tumors, IRE1 activation is independent on its expression level, especially considering the opposite consequence of XBP1 mRNA splicing and RIDD activity. In fact, altered XBP1 expression and RIDD activity has been extensively analyzed in several human cancers with controversial results since both pro-tumorigenic and tumor-suppressor activity of IRE1α have been reported (Blazanin et al., 2017; Bujisic et al., 2017; Logue et al., 2018). As an example, in glioblastoma multiform (GM), higher IRE1α activity is associated to poor prognosis due to XBP1-dependent promotion of angiogenesis and macrophage recruitment (Lhomond et al., 2018). Accordingly, higher XBP1 splicing has been found in several hematological malignancies and solid tumors as a negative prognostic factor (Chen et al., 2014; Yang et al., 2015; Xia et al., 2016; Sun et al., 2018). In TNBC, XBP1 hyperactivation promotes an adaptive response to ER and hypoxic stress through the regulation of hypoxia-inducible factor 1α (HIF1α) leading to enhanced cancer cell survival and decreased patient survival, as well as promoting TNBC resistance to paclitaxel and doxorubicin treatments (Chen et al., 2014; Hu et al., 2015). In addition, XBP1 was found to contribute to oncogenicity in prostate cancer and TNBC through the regulation of c-myc expression (Zhao et al., 2018; Sheng et al., 2019).

All these data indicate an oncogenic role for XBP1, though evidence shows a tumor-suppressor function of RIDD activity. *De facto*, RIDD activation in GM is associated with inhibition of angiogenesis and cell migration, and a longer survival of patients compared to tumors with higher XBP1 and lower RIDD activity showing more aggressiveness and a mesenchymal phenotype (Lhomond et al., 2018). Moreover, in another *in vitro* model of Ras-driven cancer, XBP1 was found to promote survival, whereas RIDD was found to suppress either survival or transformation of keratinocytes (Blazanin et al., 2017).

Altogether, these data suggest a dual role of IRE1 RNase function in tumor development depending on the prevalence of XBP1 splicing or RIDD activity. Currently, no works have analyzed the localization of IRE1 in cancer cells, in particular at MERCs, although as we have previously discussed, MERCs play an essential role in the regulation of the pro-survival or pro-apoptotic outputs of IRE1 RNase activity. In this scenario, we consider it very plausible that in cancer cells, MERCs could influence IRE1 oncogenic or tumor-suppressor activity. Moreover, the MERC modulation of IRE1α activity could be an important strategy to develop anticancer therapy. Indeed, the dual opposite outputs of IRE1 RNase activity represent a great challenge in defining the right therapy for XBP1 splicing inhibition and to promote RIDD activity, depending on the cancer type. In this view, the control of IRE1 localization at MERCs in cancer cells could represent an additional therapeutic approach. An important work showed that the IRE1α RNase inhibitor MKC8866 reduced the synthesis and secretion of protumorigenic cytokines in TNBC cells and increased paclitaxel-mediated tumor suppression in xenograft mouse models of TNBC (Logue et al., 2018). In these circumstances, *in vitro* mammosphere formation was also reduced, suggesting an important inhibitory effect of MKC8866/paclitaxel therapy on the proliferation of cancer stem cells. Accordingly, XBP1 expression was correlated to cancer stem cell expansion and tumor relapse post therapy (Chen et al., 2014).

Considerable evidence has revealed that ER chaperones such as GRP78/BiP, GRP75, CLNX, and ER oxidoreductases, such as σ1R or Ero1α, are components of MERCs (Anelli et al., 2012; Lynes et al., 2012; Rizzuto et al., 2012; Sassano et al., 2017), and all these ER stress factors are central players in the control ER–mitochondria calcium flux (Simmen et al., 2010). In the following sections, we discuss the role of MERC-located chaperones and their impact in tumorigenesis.

## Pro-Tumorigenic Function of σ1R During Regulation of Ca^2+^ Homeostasis at Mitochondria–ER Contacts

**FIGURE 3:**
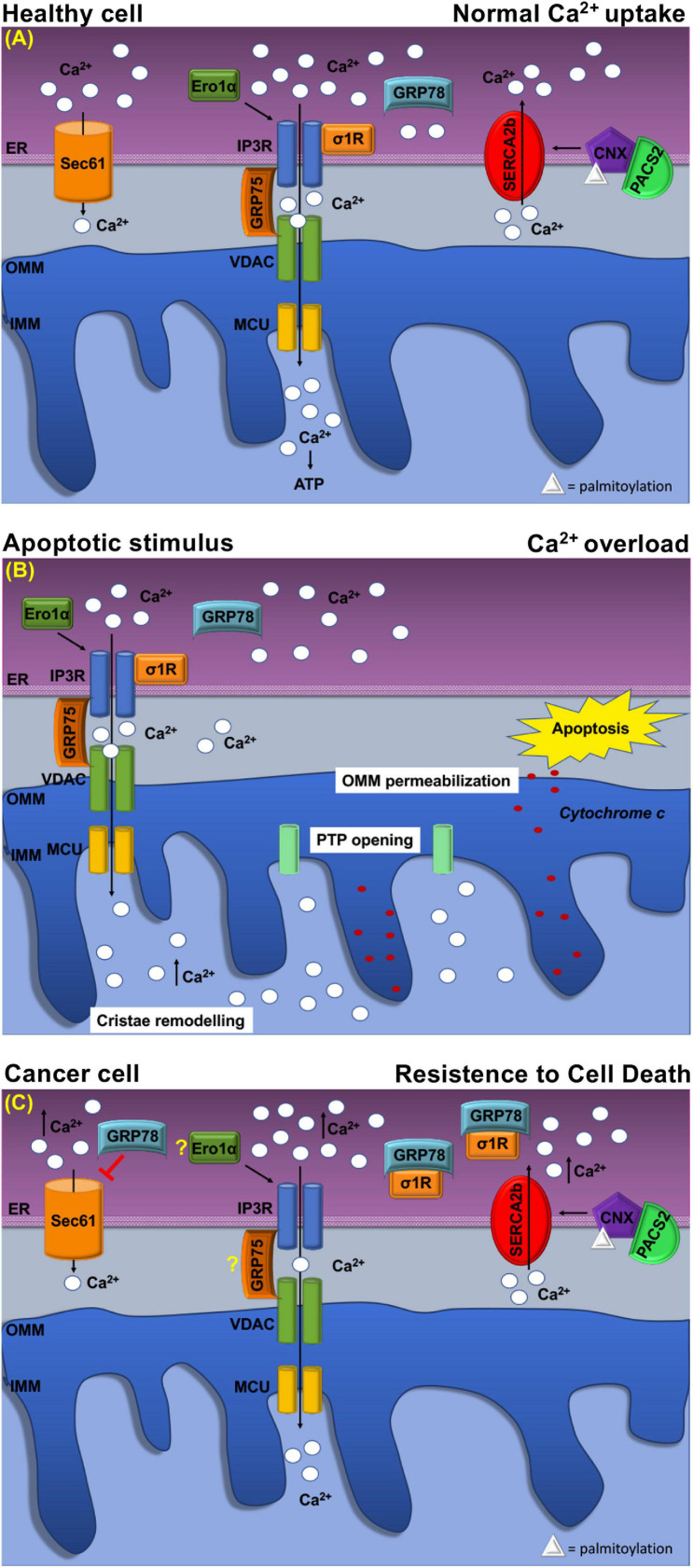
Schematic representation of Ca^2+^ handling at MERCs in healthy cell and cancer cell. **(A)** Healthy cell: The ER Ca^2+^ load is determined by ER Ca^2+^ uptake systems sarco-ER Ca2 + transport ATPases (SERCAs) and Ca^2+^-binding protein calnexin (CLNX), which is targeted to MERCs following a phosphofurin-acidic cluster sorting protein 2 (PACS2)-dependent palmitoylation to maintain high Ca^2+^ levels with the ER. Ca^2+^ is released from the ER through the activation of inositol 1,4,5-tris phosphate receptors (IP3Rs) Ca^2+^ channels that are physically connected to voltage-dependent anion channel (VDAC) located at the outer mitochondrial membrane (OMM) via glucose-regulated protein 75-kDa (GRP75). The Ca^2+^ ions transferred through the IP3Rs–GRP75–VDAC complex are imported into the mitochondrial matrix via the mitochondrial Ca^2+^ uniporter (MCU) complex. The activity of IP3Rs is controlled by several chaperones. The oxidoreductase ER oxidoreductin 1a (Ero1α) interacts with IP3Rs and modulates Ca^2+^ flux in a redox-sensitive manner. In addition, the σ1R is released from the Ca^2+^-dependent chaperone GRP78/BiP and promotes prolonged ER calcium release by stabilizing IP3R3. Regulation of Ca2 + efflux from ER is regulated, also, through the Sec61 channel that acts as a passive ER calcium leak channel. **(B)** Mitochondrial Ca^2+^ uptake affects cell death pathways. Following apoptotic stimuli, at MERCs, calcium uptake by the IP3R–GRP75–VDAC complex favors Ca^2+^ overload that promotes OMM permeabilization, inner mitochondrial membrane (IMM) cristae remodeling, and the opening of the permeability transition pore (PTP). This causes a rapid collapse of the membrane potential and the swelling of mitochondria, with consequent loss of cytochrome c that is released into the cytosol to trigger apoptosis. **(C)** At MERCs, ER chaperones regulate the Ca^2+^ homeostasis. Overexpression of GRP78/BiP at MERCs increases the Ca^2+^ storage capacity of the ER and attenuates apoptosis. Red arrows indicate inhibitory effect of GRP78/78 on the passive Ca^2+^ efflux through the Sec61 channel. Under chronic ER stress, involving prolonged ER Ca^2+^ depletion, σ1R translocates to the peripheral ER and attenuates cellular damage; in this way, σ1R is no more able to stabilize IP3R3, thereby preventing cell death. CLNX could be considered as a novel biomarker as its upregulation is related to the increased activity of sarco/endoplasmic reticulum Ca^2+^ATPase (SERCA) pump, that concentrates Ca^2+^ into the ER lumen, allowing resistance to cell death. Ero1-α and GRP75 contribution to the tumorigenesis is still unclear.

The sigma-1 receptor, σ1R, is a 26-kDa integral ER membrane protein found in several tissues (Tesei et al., 2018). Many studies showed that σ1R is located at MERCs where it forms a complex with GRP78/BiP to modulate Ca^2+^ signaling between the two organelles (Hayashi and Su, 2007; Tesei et al., 2018). Following ER Ca^2+^ release, σ1R dissociates from GRP78/BiP and binds to IP3Rs (Figure 3A), which is thus stabilized because this interaction prevents IP3Rs degradation and prolongs mitochondria Ca^2+^ uptake (Hayashi and Su, 2007; Rizzuto et al., 2012).

Evidence suggests that deregulation of σ1R may be involved in several events including apoptosis resistance, tumor growth, migration, invasive potency, angiogenesis, and cell response to the microenvironment (Crottes et al., 2013). Indeed, σ1R overexpression occurs in distinct types of cancer (Renaudo et al., 2004; Crottes et al., 2013; Soriani and Kourrich, 2019). In breast and colon cancers, σ1R overexpression occurs predominantly at the ER–mitochondria interface and correlates with an invasive and metastatic phenotype, whereas low expression levels are found in normal cells (Aydar et al., 2006; Gueguinou et al., 2017). In these cancer models, σ1R forms a molecular platform with calcium-activated K^+^ channel SK3 and Orai1, which is responsible for increased Ca^2+^ entry that finally enhances cancer cell migration (Gueguinou et al., 2017). These finding supports the pro-tumorigenic function of σ1R, which is related to the regulation of Ca^2+^ dynamics at the ER–mitochondria interface. Instead, under prolonged ER stress and Ca^2+^ depletion, σ1R translocates from MERCs to the peripheral ER (Figures 3A,C) and attenuates cellular damage, thereby preventing cell death (Hayashi and Su, 2003). Furthermore, σ1R expression is under the transcriptional control, the PERK pathway of the UPR. Indeed, ATF4 binds σ1R gene promoter, while its silencing reduced σ1R expression (Mitsuda et al., 2011) (Figure 2A,iv). In addition, σ1R itself can modulate the UPR through the modulation of IRE1α stability at MERCs (Mori et al., 2013) (Figure 2B,i) confirming the importance of the UPR for the MERC function.

## Glucose-Regulated Protein 78-kDa/Binding Immunoglobulin Protein: Ca^2+^ Handling at Mitochondria–ER Contacts and Resistance to Cell Death

GRP78/BiP has a key role in several cancers where it is often upregulated (Ibrahim et al., 2019). Higher GRP78/BiP expression is described in colon cancer, and GRP78/BiP downregulation increases epirubicin-induced apoptosis and cell migration (Xing et al., 2006; Chang et al., 2015). Similarly, in breast cancer, higher GRP78/BiP expression promotes drug resistance reducing the sensitivity of gemcitabine and causing apoptosis inhibition (Xie et al., 2016). GRP78/BiP activity depends on its intracellular localization (Casas, 2017) as sub-mitochondrial fractionation revealed that GRP78/BiP is associated with co-chaperones known to be involved in Ca^2+^-mediated signaling between the ER and mitochondria (Sun et al., 2006). Therefore, at MERCs, GRP78/BiP controls the efflux of Ca^2+^ ions from the ER by closing the Sec61 channel (Figure 3A) in the absence of translocation (Casas, 2017). However, GRP78/BiP overexpression can increase ER Ca^2+^ storage capacity and attenuate oxidant-induced Ca^2+^ fluctuations and subsequent apoptosis (Figure 3B). Additionally, GRP78/BiP silencing increases intracellular free Ca^2+^ in response to hydrogen peroxide, thereby supporting the role of GRP78/BiP in maintaining cellular Ca^2+^ homeostasis and preventing Ca^2+^-induced apoptosis (Aoki et al., 2001; Lamb et al., 2006). Finally, these considerations highlight that GRP78/BiP, at MERCs, is subjected to cancer-specific variations, but further studies are necessary to unveil the exact link among this chaperone in MERCs and tumorigenesis.

## Role of Glucose-Regulated Protein 75-kDa in Ca^2+^ Homeostasis at Mitochondria–ER Contacts

The interaction between the IP3Rs, VDAC, and GRP75 has long been proven. At the MERCs, the IP3R/VDAC1 interaction is modulated by GRP75 acting as a bridge to allow Ca^2+^ transfer from the ER to the OMM, from which the ions can easily reach the mitochondrial matrix through the mitochondrial Ca^2+^ uniporter MCU (Figure 3A). Silencing of GRP75 abolishes the IP3Rs/VDAC1 coupling, thereby reducing mitochondrial Ca^2+^ uptake in response to agonist stimulation (Rizzuto et al., 2012; Sassano et al., 2017). IP3Rs/VDAC1 interaction directly enhances Ca^2+^ accumulation in the mitochondria, while GRP75 gene silencing inhibits Ca^2+^ flux signaling at MERCs (Szabadkai et al., 2006). With this regard, GRP75 represents an important player in the control of cell fate and pathogenesis, as it is often overexpressed in different tumor types (Dundas et al., 2005; Wadhwa et al., 2006) in which it is an important regulator of tumor growth and survival. It has been remarked that, several cancer cells, in which GRP75 is increased, acquire the ability to form tumors in Balb/c nude mice, while GRP75 overexpression is sufficient to increase malignancy of breast cancer cells (Wadhwa et al., 2006). Similarly, upregulated GRP75 in human medullary thyroid cancer (MTC) tissues is essential for MTC cell survival and proliferation (Starenki et al., 2015). On these grounds, GRP75 upregulation contributes significantly to tumorigenesis, but whether this function depends on its ability to regulate Ca^2^^+^ homeostasis at MERCs requires to be further investigated. However, both the location and the function at MERCs pinpoint GRP75 as a promising target in cancer therapy.

## Role of Calnexin as Modulator of Endoplasmic Reticulum–Mitochondria Ca^2+^ Signaling and Apoptosis

Calnexin participates to the ER QC by retaining newly synthesized glycoproteins until they reach their correct conformation. At MERCs, CLNX enrichment depends on the interaction with cytosolic phosphofurin-acidic cluster-sorting protein 2 (PACS-2), which binds non-phosphorylated CLNX to the cytosolic cluster of acidic amino acids and places CLNX at MERCs (Figure 3A) (Myhill et al., 2008). Moreover, to be localized at MERCs, CLNX needs to be palmitoylated, suggesting that special lipid constitution determines MERC targeting (Raturi and Simmen, 2013).

However, the task of MERCs-localized CLNX is to regulate the activity of SERCA2b pump (Rizzuto et al., 2012). As shown in Xenopus oocytes (Roderick et al., 2000), CLNX/SERCA2b interaction requires CLNX phosphorylation on S562 in the cytosolic domain, by ERK and PKC kinases. In this state, CLNX is free to interact with the COOH terminus of SERCA2b and promotes the SERCA2b pump activity (Roderick et al., 2000).

Another role of CLNX concerns the modulation of cell sensitivity to apoptosis, which occurs differently from the other ER resident chaperones, since CLNX deficiency lowers ER luminal Ca^2+^ concentrations and protects cells from apoptosis (Figures 3B,C), while overexpression of CLNX sensitizes cells to apoptosis (Nakamura et al., 2000; Arnaudeau et al., 2002). For this reason, since its deficiency could enhance responsiveness to 5-FU-based chemotherapy, CLNX was indicated as a new prognostic biomarker of low onset in colorectal cancer patients (Nakamura et al., 2000). Moreover, other *in vitro* studies showed the importance of CLNX for HTC116 colon cancer cell growth and proliferation (Ryan et al., 2016).

Altogether, the above considerations suggest a possible role for CLNX as modulator of ER–mitochondria Ca^2+^ signaling, and therefore of apoptosis, and therefore indicating this lectin as a potential therapeutic target for cancer therapy.

## Ero1α-Mediated Ca^2+^ Flux at Mitochondria–ER Contacts and Cancer Drug Resistance

Within the ER, Ero1-α is an oxidizing enzyme that, in concert with protein disulfide isomerase (PDI), promotes proper protein folding and the maintenance of the redox state.

Instead, recent findings have shown that, at MERCs, Ero1-α regulates Ca^2+^ fluxes toward the mitochondria (Figure 3A) by interacting with the ERp44 to control IP3Rs activity (Anelli et al., 2012). Furthermore, IP3Rs-assisted Ca^2+^-transport is controlled by Ero1α oxidoreductase in concert with ERp44, another multitask chaperone, which interacts with IP3Rs in order to modulate its Ca^2+^-flux properties in a redox-sensitive manner (Higo et al., 2005; Anelli et al., 2012). Therefore, the Ero1α–ERp44 axis plays an important role in regulating Ca^2+^ homeostasis at MERCs (Anelli et al., 2012). Moreover, Ero1-α is a key regulator of oxidative stress not only within the ER but also at MERCs, where it regulates calcium flux via diffusible H_2_O_2_. Indeed, ROS generated by either ER or mitochondria in the H_2_O_2_ nanodomains generated by cristae can also localize at MERCs and perturb calcium signaling (Booth et al., 2016). Moreover, overexpression of Ero1-α is frequent in various types of tumors (Shergalis et al., 2020), in particular in the aggressive and/or drug-resistant ones with a poor prognosis. It has been proven that Ero1-α-mediated functions are key events in the cell death induced by the procaspase-activating compound-1 (PAC-1), which promotes apoptosis in a variety of cancer cell types (Seervi et al., 2013). Nevertheless, as observed in breast cancer cells, Ero1-α expression correlates with the expression of programmed cell death-ligand 1 (PD-L1), while Ero1-α knockdown results in a significant attenuation of PD-L1-mediated T-cell apoptosis, suggesting a role of Ero1-α in tumor-mediated immunosuppression (Tanaka et al., 2017).

To conclude, the mechanisms by which Ero1-α expression affects the poor prognosis of tumors are still to be elucidated and should be explored in depth, particularly by evaluating the involvement of Ero1-α-mediated Ca^2+^ flux at MERCs.

## Conclusion

In the last decades, an increasing number of studies have shed light on the role of ER MCS in coordinating key cellular functions and the maintenance of cell physiology. Along with the complex analysis of MCS composition and function, the study of MERCs has emerged as a fundamental topic in the cell biology of MCSs. Among the multiple aspects analyzed, the literature discussed hereby has unveiled an additional role played at MERCs by the UPR transducers and chaperones in the orchestration of ER-to-mitochondria calcium flux, mitochondria bioenergetics, and apoptosis, and how these functions are deregulated in specific cancer settings. Taken together, the localization of PERK, IRE1α, and ER chaperones at MERCs appears to be relevant for cancer initiation and progression, and we think that it may represent one of the aspects to be deeply explored in the upcoming cancer research. Ultimately, many outstanding questions have to be answered: Which of the effects showed by UPR transducers on cancer development are dependent on their localization at MERCs? In particular, can the dual role played by the UPR transducers as tumor suppressor versus tumor promoter be specifically attributed to the ER-localized actors rather than to the MERC-localized one? Are there any properties of the MERC-localized proteins that could be related to one specific type of cancer rather than another? How do the additional functions performed by the UPR transducers and ER chaperones at MERCs affect the strength and the outcome of UPR signaling?

Future studies defining the significance of UPR protein residence at MERCs will allow to gain further insight into their role in cellular physiology and cancer development and, auspicably, to lead to breakthroughs in therapeutic strategies.

Nomenclature: ASK1, apoptosis signal-regulating kinase 1; ATF4, activating transcription factor 4; ATF6, activating transcription factor 6; BAP31, B-cell receptor-associated protein 31; BIM, B-cell lymphoma 2; BIP, binding immunoglobulin protein; CHOP, C/EBP homologous protein; CLNX, calnexin; CLR, calreticulin; COPII, coat protein complex II; CREB3L1, CAMP responsive element binding protein 3 like 1; DR5, death receptor 5; eIF2α, eukaryotic initiation factor 2α; EMT, epithelial-to-mesenchymal transition; ER, endoplasmic reticulum; ERAD, ER-associated protein degradation; ERESs, ER exit sites; ERK, extracellular signal-regulated kinases; Ero1-α, ER oxidoreductin 1; ERp44, endoplasmic reticulum resident protein 44; ERp57, ER resident protein 57; Fis1, fission 1 protein; GADD153, growth arrest- and DNA damage-inducible gene 153; GADD34, growth arrest and DNA damage-inducible gene 34; GCN2, general control non-derepressible 2; GM, glioblastoma multiform; GRP75, glucose-regulated protein 75-kDa; GRP78, glucose-regulated protein 78-kDa; HCC, hepatocellular carcinoma cell; HIF1α, hypoxia-inducible factor 1α; HRI, heme-regulated eIF2α kinase inhibitor; IMM, inner mitochondrial membrane; IP3Rs, inositol trisphosphate receptor; IRE1, inositol-requiring enzyme 1; JNK, c-Jun N-terminal kinases; LDs, lipid droplets; MAMs, mitochondria-associated membranes; MCS, membrane contact sites; MCU, mitochondrial Ca^2+^ uniporter; MDR, mechanisms of multidrug resistance; MERCs, mitochondria–ER contacts; Mfn2, Mitofusin 2; MITOL, mitochondrial ubiquitin ligase; MRP1, multidrug resistance-associated protein 1; MTC, medullary thyroid cancer; mTOR, aammalian target of rapamycin; NE, nuclear envelope; Nrf2, nuclear factor erythroid 2-related factor 2; OMM, mitochondrial outer membrane; PACS-2, phosphofurin-acidic cluster sorting protein 2; PD-L1, programmed cell death-ligand 1; PDI, protein disulfide isomerase; PERK, protein kinase RNA-like endoplasmic reticulum kinase; PKC, protein kinase C; PP2A, protein phosphatase 2; PPM1L, protein phosphatase, Mg^2+/^Mn^2+^-dependent 1L; PTP, permeability transition pore; PTPIP51, protein tyrosine phosphatase-interacting protein-51; PUMA, p53 upregulated modulator of apoptosis; QC, quality control; RIDD, regulated Ire1-dependent decay; ROS, reactive oxygen species; S1P, site-1 protease; S1T, SERCA1 truncated proteins; SCAF, super complex assembly factor; SERCA1, sarco/endoplasmic reticulum Ca^2+^ ATPase1; SERCA2b, sarco/endoplasmic reticulum Ca^2+^ ATPase2b; σ1R, sigma-1 receptor; SIMH, stress-induced mitochondrial hyperfusion; SK3, small conductance calcium-activated potassium channel 3; TNBC, triple negative breast cancer; TRAF2, TNF receptor-associated factor 2; Trb3, Tribbles ortholog 1; TXNIP, thioredoxin-interacting protein; UPR, unfolded protein response; VDAC, voltage-dependent anion channel; VEGF, vascular endothelial growth factor; XBP1, X-box binding protein 1.

## Author Contributions

GA and VP performed the literature searches and wrote the manuscript. OM gave suggestions and significantly refined the manuscript. PR contributed to the conceptual idea, supervised the writing, and reviewed the manuscript. All authors contributed to the article and approved the submitted version.

## Conflict of Interest

The authors declare that the research was conducted in the absence of any commercial or financial relationships that could be construed as a potential conflict of interest.
